# Fixed and dynamic predictors of treatment process in therapeutic communities for substance abusers in Belgium

**DOI:** 10.1186/1747-597X-7-43

**Published:** 2012-10-11

**Authors:** Ilse Goethals, Wouter Vanderplasschen, Stijn Vandevelde, Eric Broekaert

**Affiliations:** 1 Faculty of Psychology and Educational Sciences, Department of Orthopedagogics (Special Education), Ghent University, Henri Dunantlaan 2, 9000, Ghent, Belgium; 2 Faculty of Psychology and Educational Sciences, Department of Orthopedagogics (Special Education), Ghent University, Henri Dunantlaan 2, 9000, Ghent, Belgium; 3 Faculty of Education, Health and Social Work, Department of Orthopedagogics, University College Ghent, Voskenslaan 362, 9000, Ghent, Belgium; 4 Faculty of Psychology and Educational Sciences, Department of Orthopedagogics (Special Education), Ghent University, Henri Dunantlaan 2, 9000, Ghent, Belgium

**Keywords:** Therapeutic communities, Treatment process, Motivation, Psychological distress, Demographics

## Abstract

**Background:**

Research on substance abuse treatment services in general reflects substantial attention to the notion of treatment process. Despite the growing popularity of process studies, only a few researchers have used instruments specifically tailored to measure the therapeutic community (TC) treatment process, and even fewer have investigated client attributes in relation to early TC treatment process experiences. The aim of the current study is to address this gap by exploring clients’ early in-treatment experiences and to determine the predictors that are related to the treatment process, using a TC-specific multidimensional instrument.

**Methods:**

Data was gathered among 157 adults in five TCs in Flanders (Belgium). Descriptive statistics were used to explore clients’ early in-treatment experiences and multiple linear regressions were conducted to determine the fixed and dynamic predictors of Community Environment and Personal Development and Change (two indicators of TC treatment process).

**Results:**

Clients reveal a more positive first-month response to TC social processes than to personal-development processes that require self-reflection and insight. The variance in clients’ ratings of Community Environment was primarily due to dynamic client factors, while the variance in clients’ ratings of Personal Development and Change was only related to fixed client factors. Suitability for treatment was the strongest predictor of Community Environment ratings, whereas a judicial referral more strongly predicted Personal Development and Change scores.

**Conclusions:**

Special attention should be devoted to suitability for treatment as part of motivational assessment as this seems to be a very strong predictor of how clients react to the initiation stage of TC treatment. To help improve clients’ (meta-)cognitive skills needed to achieve insight and self-reflection and perhaps speed up the process of recovery, the authors suggest the introduction of (meta-)cognitive training strategies in the pre-program and/or the induction stage of a TC program.

## Background

The therapeutic community for addictions (TC), developed in the early 1960s, is a widely used treatment modality for people with severe substance abuse problems. The TC model is based on the view that substance abuse is a disorder that involves the whole person. Therefore, the main goal of TC treatment is to change one’s lifestyle and identity through mutual help and self-help. Unlike other treatment modalities, the social environment itself is the treatment [1].

Since their inception, researchers have evaluated the effectiveness of TC treatment programs, showing a positive correlation between retention, often indicated as the time spent in treatment, and post-treatment substance use and criminal involvement rates [2-6]. Despite these promising results, studies also revealed high dropout rates [7-11], which may differ greatly between TC programs [12,13]. Since dropout is associated with poor treatment outcomes, and since considerable variations exist between TC treatment programs, researchers aimed to define and measure the TC treatment process [1,14-18].

### Measuring TC treatment process

Analyzing the TC treatment process appeared to be a challenging endeavor. Notably, early research efforts concentrated on the treatment structure – such as the description of programs or of the treatment provided [19,20] – or on client attributes in relation to retention [15,21]. During the last decade, new areas of investigation have begun to emerge that focused more on the black box of treatment [22]. In this respect, a limited number of TC studies have investigated hypotheses concerning the sequential links between client attributes (e.g. background variables, motivation and readiness for treatment), treatment engagement (e.g. treatment participation and therapeutic relationship) and retention (e.g. time in treatment) or treatment outcomes [23-27].

Despite the promising accomplishments of these studies for theory and practice, it has been noted that these investigations did not use TC-specific instruments and so did not entirely capture the effective ingredients of the TC process. As a response to this limitation, Phoenix House Foundation, one of the largest TC organizations in the United States, engaged the RAND corporation to identify and operationalize the essential elements of the community-as-treatment process, as described by De Leon [1]. This resulted in an assessment tool called the ‘Dimensions of Change Instrument’ (DCI) [28]. The DCI is a self-report questionnaire that measures the TC treatment process based on two core dimensions of effective agency: (1) Community Environment and (2) Personal Development and Change. The first dimension assesses client’s perception of the environment and the interactions in that environment that provide opportunities for mutual help and self help. The second dimension measures client’s psychological and cognitive skills which are mediated by the interactions in the community environment. The underlying assumption is that the Community Environment facilitates and promotes the interactions of clients and staff, which fosters client’s insight, understanding and change. The degree of Personal Development and Change in turn impacts on the nature and scope of the interactions which – in their turn – can have effects on the Community Environment. In contrast to other treatment process models, Personal Development and Change is not characterized as a proximal outcome but rather as an integral part of the process [28].

Hypotheses concerning client progress and treatment retention have been tested in diverse populations while using the DCI. Findings show constant improvements over time in the client-level treatment process for adult as well as adolescent treatment samples [29]. During the early stages of treatment, adults’ perception of the treatment process and adolescents’ view on personal development and change predict retention in the subsequent stage [30-32].

Examination of the effect of client variables on the TC treatment process revealed higher community environment scores for adult clients who were 25 years or older, female, and had a prior drug treatment experience. Adolescents with two or more arrests in the two years prior to admission had lower scores on both process dimensions [33]. In prison settings, clients who where older or poly-substance users had better community environment scores, while prisoners with children and fewer lifetime arrests had better scores on Personal Development and Change [34].

### Fixed and dynamic predictors of TC treatment process

The variables associated with the treatment process in previously mentioned studies are demographic or background variables which are often referred to as fixed client characteristics. Yet other variables that need to be considered as determinants of the treatment process are dynamic client-level variables which describe the changing or client perception variables [35] such as client motivation, treatment readiness and psychological well-being. Empirically, motivation and readiness for treatment have proved to be strong predictors of both clients’ early responses to TC treatment [23,36] and treatment retention [37]. Moreover, it has been suggested that dynamic variables have a stronger predictive value than socio-demographic or background variables [26,35]. Also, theoretically it might be assumed that a client’s perception of the TC treatment process is affected by dynamic client factors. For example, we can assume that a client with a low level of motivation or a client who is not ready for a long-term residential treatment will not fully engage in the therapeutic activities, nor will he/she take responsibility for others in the program [1]. Motivation will also determine the degree to which a client recognizes the extent of his/her problems [38] and whether he/she is committed to self-management and a drug-free lifestyle [1]. But also feelings of psychological distress can affect a client’s perception on the TC treatment process. For instance, a hostile client will have issues of trust and greater difficulties connecting with peers, where a depressed client will show a diminished interest in the TC’s daily activities [1].

### Aims of this study

Despite the fact that dynamic client variables as well as fixed client variables have been studied amongst substance users in seeking, complying with and remaining in treatment, little still is known about their relationship with the TC treatment process in particular. Yet, finding out how individuals with different needs and experiences react to ‘community as method’ will help identify clients needs and point to those aspects of the TC methodology that could be improved.

Consequently, the overall goal of the present study is to explore in depth the association of these variables with the treatment process as measured by the DCI. First, we will investigate clients’ early perceptions of the TC treatment process, analyzing the mean scores on the eight DCI subscales. Secondly, we will determine the fixed and dynamic predictors related to Community Environment and Personal Development and Change (e.g. two indicators of the treatment process). Finally, analysis should reveal whether or not dynamic client-level variables are better predictors of the treatment process indicators than fixed client variables.

## Methods

### Participants

The study was carried out in the five hierarchical concept-based therapeutic communities for addictions in Flanders, Belgium. The five programs range in capacity from 14 to 30 clients with lengths of stay between 10 and 18 months. The participants were selected from a cohort of 180 substance abusers who started treatment in one of these five long-term residential facilities between March 2009 and April 2011. Eligibility for this study entry required that participants were 18 years of age or older, had no prior experience with TC treatment and had sufficient knowledge of the Dutch language.

Data from 157 respondents (87% of the total study sample) who stayed in treatment long enough to be eligible to complete the first in-treatment assessment (>15 days; see Table 1 for demographic and background characteristics) were used for the present study.

**Table 1:** Frequencies and percents of client characteristics

**Characteristics**	**n**	**(%)**
**Gender**		
Male	132	(84)
Female	25	(16)
**Race/ethnicity**		
Caucasian	148	(97)
Other	9	(3)
**Marital status**		
Single	141	(90)
Married	5	(3)
Divorced	11	(7)
**Education level (n = 155)**		
Primary to lower secondary Education	25	(16)
Vocational certification	90	(57)
Secondary to higher Education	42	(27)
**Primary drug problem**		
Amphetamine	34	(22)
Cocaine	29	(19)
Heroin	61	(39)
Marijuana	15	(10)
Other	18	(11)
**Legal reference**		
Yes	64	(41)
No	93	(59)
**Prior drug treatment**		
Yes	137	(86)
No	20	(13)
**A drug/alcohol abusing parent**		
Yes	75	(48)
No	81	(52)

### Procedure

Approximately one to two weeks before entering the TC, participants were asked to take part in a face-to-face interview. Information was gathered about socio-demographic background, physical and psychological health, education, employment, substance abuse history, illegal activities and family/social relationships. In-treatment assessment took place 30 days after the initial interview, gathering information on treatment process variables, psychological well-being, personality disorders and motivation. This particular time frame was chosen to minimize the effect of maturation. More specifically, as two of the five participating TCs have a welcome phase, where clients are prepared for a TC life over a period of one to two months, it is assumed that these clients will have a different perspective on TC treatment than clients who enter a TC program relatively unprepared. To be able to take into account this maturation effect, we decided that baseline data would be gathered at the moment the client decided to enter a TC program. For clients that were in a welcome phase, this moment was set around 10 to 14 days before intake. The unprepared clients – clients who entered a TC after being in a detoxification center or a crisis center, or who came straight from prison – were assessed 1 to 5 days before intake.

While most of the data was gathered by the main researcher, some EuropASI data was also collected with the help of professionals or master students in Educational Sciences, trained in EuropASI interviews. The participants were informed that the data would be processed anonymously and that the overall purpose of the study was to assess those aspects of TC treatment which might be improved. Written informed consent was obtained from each participant prior to the first interview. Ethical approval for the study was granted by the Ethical Review Board of the Faculty of Psychology and Educational Sciences at Ghent University.

### Instruments

*Client background data, demographic data and the severity of substance use and related problems* were obtained with the EuropASI, an adapted and validated version of the Addiction Severity Index (ASI) for the European context [39,40]. The ASI explores clients’ current and lifetime functioning in seven different areas (medical status; employment and support; drug use; alcohol use; legal status; family and social relationships; and psychiatric status), displaying a multidimensional problem severity profile. An ASI composite score is calculated for each of the seven life domains (range 0–1), with higher scores indicating higher problem severity [41]. In our study, composite scores are based on events that occurred 30 days before entering a detoxification centre and on the client’s perceived need for help at that time. However, for clients who entered the TC following a period of imprisonment or hospitalisation, the composite scores are based on the events that occurred 30 days prior to TC intake.

*Treatment motivation* was measured with the Circumstances Motivation Readiness and Suitability Scales (CMRS) [15]. This is a self-administered questionnaire with 42 Likert-type items rated on a 5-point scale, which ranges from ‘strongly disagree’ to ‘strongly agree’. The instrument’s first scale, ‘Circumstances*’*, refers to the external conditions or reasons that influence people to enter or leave treatment. The second scale, ‘Motivation*’* (internal pressures), refers to the individual’s inner reasons for change. These reasons can be initiated by feelings of guilt or self-loathing, i.e. negative feelings that are associated with a drug-related lifestyle, or by a belief in one’s own personal growth and the desire for a better life. The third scale, ‘Readiness*’*, underlines the perceived need for treatment in order to change. The *‘*Suitability*’* items examine the individual’s perception of the appropriateness of the treatment modality. This scale determines to what extent clients think the TC treatment matches their needs. The psychometric properties of the Dutch translation were found to be acceptable and in line with the findings of the American studies [42]. The current study obtained Cronbach alpha coefficients ranging from .67 to .83 across the four scales.

*Psychological distress* was measured with the Brief Symptom Inventory (BSI) [43,44], derived from the SCL-90-R (Symptom Check List-90-R). This is a 53-item self-report scale used to measure recent psychological complaints (past 7 days) (somatization, obsessive-compulsive behavior, interpersonal sensitivity, depression, anxiety, hostility, phobic anxiety, paranoid ideation and psychoticism). Symptoms are rated on a 5-point Likert scale ranging from ‘Not at all’ to ‘Extremely’ (range 0–4). The higher the score, the greater the level of psychological distress. In this study, we used the Global Severity Index (GSI), an average rating of all 53 items and overall score of psychological functioning (Cronbach Alpha of .95). The cut-off score of the GSI (0.66 for males; 0.71 for females) is used as a general measure of psychopathology.

To measure *personality traits* the Assessment of DSM-IV Personality Disorders (ADP-IV) questionnaire was used [45]. The ADP-IV is a validated Dutch self-report measure consisting of 94 Likert-type items that allows for a categorical and dimensional assessment of the 12 DSM-IV personality disorders [46,47]. The dimensional interpretation emphasizes the continuity between normality and pathology of the DSM-IV personality ‘traits’ and is measured on a 7-point Trait (T) scale. The ‘distress’ of the subject or his/her environment as a consequence of having the trait criterion is assessed with a 3-point distress (D) scale. The categorical diagnostic evaluation is based on the following algorithm: ‘T > 4 and D > 1’; an item is scored ‘pathological’ when the trait score is larger than four and the distress score is larger than one. In accordance with the DSM-IV criteria, four or more items need to be scored positive/pathological before a diagnosis of a personality disorder can be made [45].

For the bivariate and multivariate analysis we used the dimensional assessment by summing the ADP-IV trait scores for the 3 clusters. Cluster A represents disorders that are marked as ‘odd or eccentric behavior’: paranoid, schizoid and schizotypical personality disorders. Cluster B refers to those disorders that manifest ‘dramatic, emotional or erratic behavior’, i.e. antisocial, borderline, narcissistic and histrionic personality disorders. Finally, Cluster C corresponds to disorders that are marked as anxious or fearful behavior, i.e. avoidant, dependent and obsessive-compulsive personality disorders. The Cronbach’s alpha coefficients ranged from .85 to .88 across the three clusters.

The *treatment process* was assessed with the Dimensions of Change Instrument (DCI) (cf. background). It is a 54-item questionnaire that assesses clients perceptions on various components of the TC treatment process. All items are positively worded and ask respondents to indicate their extent of agreement on a 5-point scale (1 = *Not at all* to 5 = *Completely*) with higher scores indicating a greater extent of agreement. The instrument consists of eight different subscales [28]. These are: (1) *Community Responsibility (CR) –* the client personally accepts the rules of conduct; *(2) Clarity and Safety (CS)* – the client has a good understanding of the goals, structure, patterns of interpersonal interaction and feels safe in the community environment; (3) *Group Process (GP)* – the client observes the group meetings as helpful and perceives that residents actively participate in group therapy activities; (4) *Resident Sharing, Support, and Enthusiasm (RS)* – the client perceives residents as being enthusiastically engaged in sharing of personal feelings and being supportive in social interactions; (5) *Introspection and Self-Management (IS) -* the client engages in personal self-awareness and reflection, and adopts self-management enhancement activities; (6) *Positive Self-Attitude and Commitment to Abstinence (PS) –* the client admits to feelings of self-efficacy and commitment to achieving abstinence; (7) *Problem Recognition (PR)* – the client recognizes that his/her personal behavior and attitudes can lead to personal and interpersonal problems; (8) *Social Network (SN)* – the client believes he or she has a supportive social network outside of the TC community) [31]. The first four subscales are clustered in the *Community Environment (CE)* summary dimension whereas the latter four are grouped in the ‘Personal *Development and Change (PDC)* summary dimension.

For the present study, the instrument has been translated into Dutch using back and forward translation. Subscale scores were calculated as the mean of the respective items, while summary scores for the two DCI dimensions represent the mean scores of the respective subscales. The internal consistency of the Dutch version of the DCI shows alpha reliability coefficients of .87 for the Community Environment summary dimension and .82 for the Personal Development and Change summary dimension. For the separate scales the Cronbach’s alpha ranges from .61 to .81.

### Data-analysis

To verify clients’ early perceptions on the TC treatment process we computed the means and standard deviations for the two DCI summary dimensions and the eight DCI subscales. Multiple linear regressions were used to determine the fixed and dynamic predictors for the two DCI summary dimensions. We first used bivariate analyses, including Pearson product–moment correlations, independent t-tests and one-way ANOVA’s, as appropriate to the level of measurement, to determine any relations between potential predictor variables and the two DCI summary dimensions, or at least one of the dimension’s subscales. These tests were performed on treatment site, client demographics (e.g. age, gender and ethnicity), all important EuropASI items, the EuropASI composite scores, the three ADP-IV clusters, the BSI total average score, and the four scales of the CMRS. To help control for the inflated alpha levels due to multiple testing and to focus results on the larger effect sizes for clinical significance, we only withheld the variables that were associated at the 0.01 level of significance.

Each time, the variables were entered in the regression equation in one single step using the default method. Data analysis was conducted using the SPSS 19.0 statistical program. Visual examination of the standardized residuals (the errors) by the regression standardized predicted values indicate that both, the assumption of linearity and homoscedasiticty, was met; the residual plot was rectangular with concentration of points around zero, respectively. Also the collinearity diagnostics revealed no difficulties. Variance inflation factors (VIF) and tolerance values were within the acceptable ranges; all VIF values were below 10.0 and all tolerance values were above 0.10.

## Results

### Study sample characteristics

Tables 1 and 2 give an overview of client characteristics for the sample that completed the in-treatment assessment. Due to missing data, the number of residents for whom data is available varied from 155 to 157. When comparing the eligible group on EuropASI variables with the group that left treatment prematurely (n = 23) we only detected three significant differences. Those who left the TC program earlier were more likely to be divorced (21.7% vs 6.5%; χ^2^ = 6.51, *p* < 0.05) and more likely to have no diploma or a primary school diploma (39.1% vs 16. 1%; χ^2^ = 7.35, *p* < 0.05). Based on the EuropASI composite scores we also found that this group of early dropouts had significantly more (*t*(178) = −2.66; *p* < 0.01) psychological problems (*M* = 0.46; *SD* = 0.25) than the participants who stayed in treatment longer (*M* = 0.33; *SD* = 0.22).

**Table 2:** Means and standard deviations of client characteristics

**Characteristics**	**M**	**SD**
**Age**	27	(5.05)
EuropASI composite scores*		
Medical disorder	0.25	(0.28)
Employment problems	0.88	(0.28)
Alcohol problems	0.26	(0.32)
Drugs problems	0.27	(0.14)
Legal problems	0.34	(0.26)
Family relationships	0.28	(0.25)
Social relationships	0.21	(0.20)
Psychiatric disturbances	0.33	(0.22)
**Personality traits (ADP-IV)**		
Cluster A	71.66	(20.80)
Cluster B	117.78	(27.85)
Cluster C	74.49	(20,34)
**Psychological distress (BSI)**	0.92	(0.53)
**CMRS**		
Circumstances	3.96	(0.58)
Motivation	4.03	(0.48)
Readiness	4.31	(0.49)
Suitability	4.17	(0.42)

*range 0 – 1: higher score indicating higher problem severity.

Of the 157 residents, 84% were males. The age of the residents ranged from 18 to 45 with a mean age of 27. The majority of the residents were Belgian (97%), single (90%), had a vocational training (57%) and identified heroin (39%) or amphetamine (22%) as their primary drug. About 40% entered treatment with a judicial referral. Of the total sample, 30% scored above the clinical cut-off score (e.g. one or more diagnoses of DSM-IV personality disorders) for cluster B personality disorders, while 14% and 13% scored above the clinical cut-off score for cluster A and C personality disorders, respectively. More than 75% of the study sample reported medium high (between the mean and + 1SD) to high motivational scores (+ 1SD or more above the mean) on the CMRS subscales ‘Circumstances’ (74%) and ‘Readiness’ (81%). Fewer participants scored medium high (between the mean and + 1SD) to high (+ 1SD or more above the mean) on the subscales ‘Suitability’ (59%) and ‘Motivation’ (54%). Finally, 68% of the study sample had a score above the clinical cut-off score (0.66 for males; 0.71 for females) for overall psychological distress.

### Perceptions of the treatment process

Approximately 15 to 30 days after admission to a TC in Flanders, clients appear to have a slightly more positive attitude towards elements of the Community Environment than towards their own Personal Development and Change process (see Table 3). Within the Community Environment summary dimension we notice the highest average score for the subscale ‘Community Responsibility’, and the lowest average score for the subscale ‘Group Process’. Regarding the Personal Development and Change summary dimension, we found the highest average score for the subscale ‘Positive Self-attitude and Commitment to Abstinence’ and the lowest average score for the subscale ‘Problem Recognition’.

**Table 3:** Means and standard deviations of the DCI dimensions and subscales

**DCI**	**M**	**(SD)**
**Community Environment (CE)**	**3.80**	**(0.48)**
Community responsibility (CR)	4.09	(0.66)
Clarity and Safety (CS)	4.00	(0.59)
Group Process (GP)	3.55	(0.63)
Resident Support, Sharing and Enthusiasm (RS)	3.82	(0.56)
**Personal Development and Change (PDC)**	**2.98**	**(0.44)**
Introspection and Self-Management (IS)	3.29	(0.65)
Positive self-attitude and commitment to abstinence (PS)	3.61	(0.66)
Problem recognition (PR)	2.84	(0.85)
Social Network (SN)	3.55	(0.97)

**Figure 1:**
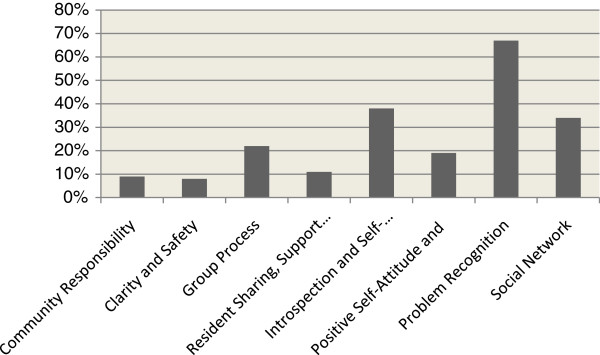
Proportion of respondents reporting low scores for the eight DCI subscales.

While the mean scores provide an overall picture of the total sample, Figure 1 shows the proportion of clients reporting low scores on the DCI subscales (score < 3). The results are divergent between the subscales, consisting in particular of a large number of subjects with a low score for ‘Problem Recognition’ (66.9%). Also, more than 30% of the clients had a low score on ‘Introspection and Self-management’ (37.6%) and ‘Social Network’ (34.4%). In comparison with the other subscales, relatively few subjects reported low scores on ‘Clarity and Safety’ (7.6 %) ‘Community Responsibility’ (8.9%) and on ‘Resident Sharing, Support and Enthusiasm’ (10.8%).

### Community Environment: Bivariate analyses

The analyses identified nine client characteristics that were significantly (*p* < 0.01) associated with the Community Environment summary dimension or at least one of the four Community Environment subscales. These client characteristics were age, heroine use, having a parent with alcohol/drug problems, personality traits ‘odd and eccentric behavior’ (cluster A) and ‘dramatic, emotional or erratic behavior’ (cluster B) (e.g. five fixed client variables), psychological distress, motivation, readiness and suitability (e.g. four dynamic client variables). Independent *t*-test revealed a significant relationship between the variable ‘heroine use’ and the ‘Clarity and Safety’ subscale (*t*_(153)_ = −2.43; *p* < 0.01); clients who indicated heroine as their primary drug use showed higher ‘Clarity and Safety’ scores (*M* = 3.10, *SD* = 0.43) than the clients who did not report heroine as their primary drug use (*M* = 2.90; *SD* = 0.42). A significant association was also found for the subscale ‘Group Process’ and the variable ‘having a parent with alcohol or drug problems’ (*t*_(153)_ = −2.65; *p* < 0.01); clients who reported a drug or alcohol abusing parent showed higher Group Process scores (*M* = 3.68, *SD* = 0.64) in comparison to the clients who did not (*M* = 3.41, *SD* = 0.60). Pearson product–moment correlations for continuous variables are presented in Table 4. Overall we notice that the personality trait ‘dramatic, emotional or erratic behavior’ (Cluster B) and the four dynamic variables were significantly (*p* < 0.01) related to the Community Environment dimension and subscales. Odd and eccentric behavior (cluster A) could be linked to the summary dimension and the two subscales ‘Group Process’ and ‘Resident Sharing, Support and Enthusiasm’. Finally, age was only associated with the subscale ‘Group Process’.

**Table 4:** Pearson product–moment correlations of selected continuous variables for the DCI dimensions and subscales

	**CE**	**CR**	**CS**	**GP**	**RS**	**PDC**	**IS**	**PS**	**PR**	**SN**
**1. Age**	0.09	0.37	−0.01	0.23*	−0.00	-	-	-	-	-
**2. Odd and eccentric**	−0.22*	−0.06	−0.12	−0.24*	−0.21*	−0.22*	−0.33*	−0.37*	−0.29*	−0.18
**3. Dramatic, emotional or erratic**	−0.29*	−0.23*	−0.26*	−0.23*	−0.26*	−0.21*	−0.45*	−0.39*	0.38*	−0.12
**4. Anxious and fearful**	-	-	-	-	-	−0.20	−0.30*	−0.39*	0.20	−0.07
**5. Psychological distress**	−0.33*	−0.22*	−0.26*	−0.32*	−0.20	−0.18	−0.32*	−0.47*	0.35*	−0.08
**6. Motivation**	0.31*	0.25*	0.24*	0.24*	0.29*	0.20	0.07	0.14	0.22*	0.08
**7. Readiness**	0.42*	0.33*	0.33*	0.32*	0.34*	0.29*	0.20	0.28*	0.12	0.16
**8. Suitability**	0.56*	0.42*	0.43*	0.41*	0.45*	0.33*	0.29*	0.32*	0.18	0.07

*Denotes the variables that correlated at the 0.01 level of significance.

### Community Environment: Multivariate analyses

The regression model significantly predicted Community Environment scores (*F*_(8,142)_ = 13.93; *p* < 0.01), accounting for 47% of the variance in this dimension. Further examination revealed two fixed client variables (e.g. ‘having a parent with alcohol/drug problems’ and a personality trait of ‘dramatic, emotional or erratic behavior’ (Cluster B)) and two dynamic client variables (e.g. ‘psychological distress’ and ‘Suitability’) as significant predictors. Clients who grew up with an alcohol or drug-abusing parent and clients who reported high levels of treatment suitability reported higher Community Environment scores. On the other hand, clients who revealed higher levels of dramatic, emotional or erratic behavior, or higher levels of psychological distress, reported lower Community Environment scores. The standardized regression coefficients identified ‘Suitability’ (*β* = 0.53) and ‘psychological distress’ (*β* = −0.28) as the strongest predictors of the Community Environment dimension after statistically controlling for those variables included in the model (see Table 5).

**Table 5:** Multiple linear regression of fixed and dynamic client variables on the two DCI summary dimension

	**Community environment**			**Personal development & change**		
**Fixed variables**	***B***	***SE B***	**β**	***B***	***SE B***	**β**
Age	−0.01	0.01	-.07	-	-	-
Heroin use	0.06	0.06	.06	−0.12	0.07	-.13
Cocaine use	-	-	-	0.20	0.09	.18*
Judicial referral	-	-	-	0.25	0.07	.28**
Parent with alcohol/drug problems	0.17	0.06	.17**	-	-	-
Odd and eccentric (Cluster A)	0.00	0.00	.04	−0.01	0.00	-.07
Dramatic, emotional and erratic (cluster B)	−0.00	0.00	-.21*	−0.01	0.00	-.06
Anxious or fearfull (cluster C)	-	-	-	−0.00	0.00	.12
**Dynamic variables**						
Psychological distress	−0.46	0.14	-.28**	−0.03	0.11	-.02
Motivation	0.15	0.09	.15	0.15	0.09	.16
Readiness	−0.10	0.10	-.10	0.04	0.16	.03
Suitability	0.59	0.11	.53**	0.22	0.12	.21

*p < 0.05, **p < 0.01.

### Personal Development and Change: Bivariate analyses

The analyses identified 10 client characteristics that were significantly (*p* < 0.01) associated with the Personal Development and Change summary dimension or at least one of the four subscales. These client characteristics were heroin use, cocaine use, judicial referral, personality traits (ADP-IV) ‘odd and eccentric behavior’ (cluster A), ‘dramatic, emotional or erratic behavior’ (cluster B) and ‘anxious or fearful behavior’ (Cluster C) (e.g. six fixed client characteristics), and psychological distress, motivation, readiness and suitability (e.g. four dynamic client variables). The ‘Social Network’ subscale was significantly related to heroin use (*t*_(153)_ = 2.83; *p* < 0.01) and cocaine use (*t*_(153)_ = −2.85; *p* < 0.01); residents who specified heroine as their primary drug use showed lower Social Network scores (*M* = 3.26, *SD* = 0.94) than clients who did not (*M* = 3.70, *SD* = 0.95). Clients who reported cocaine as their primary drug use revealed higher Social Network Scores (*M* = 3.99, *SD* = 0.94) than the clients who did not specify cocaine as a primary drug of use (*M* = 3.43, *SD* = 0.95). Judicial referral was significantly related to the Personal Development and Change dimension (*t*_(153)_ = −2.76; *p* < 0.01); clients with a judicial referral – as a condition of probation or parole – reported higher scores (*M* = 3.10, *SD* = 0.42) on this dimension than clients who entered treatment voluntarily or who entered following advice from family, friends or professionals (*M* = 2.90, *SD* = 0.43). Pearson product–moment correlations for continuous variables are presented in Table 4. Interestingly, most of these variables showed a significant association with the subscale ‘Positive Self-Attitude and Commitment to Abstinence’ but none could be related to the subscale ‘Social Network’. Additionally, the subscale ‘Problem Recognition’ was significantly and positively related to two of the psychiatric traits (Clusters A and B) and the variable ‘psychological distress’ indicating higher Problem Recognition scores in clients with more pathological problems.

### Personal development and change: multivariate analyses

The regression model significantly predicted Personal Development and Change scores (*F*_(10,142)_ = 5.74, *p* < 0.001), accounting for 29% of the variance in this DCI summary dimension. Only two fixed client characteristics (‘cocaine use’ and ‘judicial referral’) were statistically significant. Clients who reported cocaine as their major problem use and clients who entered treatment based on a judicial referral reported higher Personal Development and Change scores. The standardized regression coefficients indicated ‘judicial referral’ (*β* = 0.28) as the strongest predictor of the Personal Development and Change dimension after statistically controlling for those variables included in the model (see Table 5).

## Discussions

### Clients’ early perceptions of TC treatment process

Consistent with prior research [31], findings revealed relatively high mean scores (> score 3) on the various subscales of the DCI, indicating that most clients have a positive attitude towards their first month in-treatment experiences. Furthermore, the high mean score on the DCI subscale ‘Community Responsibility’ shows clients’ early adherence to the TC program’s regime, their willingness to uphold the TC standards and ethics, and their belief that all members are equally responsible for the program to work. This finding, which has also been observed in the American DCI studies [28-31], is not surprising considering that the primary objective of the TC’s induction stage is to rapidly assimilate new residents into the community by introducing them to the cardinal rules, community regulations and procedures of the TC program. In theory, it is assumed that clients’ early adherence to the TC program might reduce the change of premature dropout [1].

The low mean score on the subscale ‘Problem Recognition’ and the large proportion of clients that reported low scores on ‘Introspection and Self-Management’ and ‘Social Network’ indicate that many clients have not yet developed a sufficient level of personal awareness and insight. This finding corresponds with the TC developmental view on recovery which states that behavioral and attitude changes mostly precede insight about the self. However, although self-reflection and insight might be less apparent in the early stages of TC treatment, they are crucial in maintaining changes and long-term recovery. Once clients recognize that inner thoughts, perceptions and feelings can cause drug seeking and other self-destructive or self-defeating behaviors, they learn how to deal with life experiences more constructively and eventually develop a sense of self-efficacy [1]. All of these changes should accordingly provide the tools to sustain recovery, even after discharge. Generally, our findings suggest that most clients will require additional interventions or services to attain the level of clinical progress needed to successfully engage in treatment. In particular, early interventions that targeting cognition, such as ‘node-link mapping’, which consists of “*drawing spatial-verbal displays to visually represent interrelationships between ideas, feelings, facts and experiences*” [48], may be necessary to increase personal development and to speed up the recovery process.

Noteworthy is the observation that in our study sample clients scored higher on Community Environment subscales whereas clients in the American study samples [28-31] scored higher on the Personal Development and Change subscales. Plausible explanations for the diversity in DCI scores might be related to differences in study sample characteristics or variations in program structure. However, comparative studies will be needed to ascertain these hypotheses.

### Determinants of Community Environment

Findings from the multivariate regression analysis revealed that while both fixed and dynamic client factors were significantly related to the Community Environment dimension, the strongest predictors were dynamic variables. The most powerful predictor was ‘suitability’. It was shown that clients who reported higher suitability scores also reported higher Community Environment scores. More precisely, clients who display the willingness to free themselves of their drug abuser identity, make changes to their earlier lifestyle and believe that a high-intensity recovery program (such as a long-term residential TC) best fits their needs, also have a more positive perspective on Community Environment during the first month of TC treatment. Several studies have shown that higher levels of motivation can be associated with early engagement in the TC treatment process [23,26,27,36]. However, close examination of the literature reveals that all of these studies on predictors of the TC process focused on motivation and readiness for treatment, rather than on treatment suitability. The main reason for this lies in the way motivation was defined and measured. Some investigators used the short version of the CMRS, which in fact excludes the items from the suitability scale [27]. Others used scales that were developed in accordance with a specific theory [36] or model [26]. Thus, based on the research literature we might conclude that evidence about the potential relationship between clients’ suitability for treatment and the TC treatment process is lacking because it has never been studied in this context before. Nevertheless, given that in our study suitability predicted a large proportion of the variance in Community Environment, while motivation and readiness for treatment were not significantly related, more attention should be given to the suitability scale in future TC treatment process studies.

The suitability-treatment process interaction contains important implications for clinical strategies and treatment policy. Clients who enter TC programs are at different levels of suitability. Treatment providers may use this knowledge to either select clients that are ready for ‘community as method’, use specific strategies to enhance treatment suitability itself, or even redirect clients to other, less demanding treatment programs. Such efforts can strengthen existing programs and also ensure more efficient use of TC resources. Specific interventions that might improve or help maintain perceptions of suitability, are motivational interviewing [49], the implementation of a separate induction or welcome stage [50] and the introduction of the Senior Professor model [51]. Introducing these interventions might be a useful strategy for exploring the individual’s understanding of the active ‘ingredients’ of the TC treatment model in order to identify and resolve discrepancies between what the client perceives and how TCs actually work.

The second strongest predictor of Community Environment process scores was the dynamic variable ‘psychological distress’. It was indicated that clients who reported higher levels of psychological distress in the course of the first month of treatment reported lower Community Environment scores. This finding is not in line with prior studies that found higher participation rates in clients with more severe psychological problems [52,53]. These researchers have argued that substance abuse treatment programs treat clients with more psychological problems more intensively than they treat clients with less psychological problems. Therefore, clients who have higher needs or feel more distressed will obtain more services. Other studies have shown that the presence of psychological problems does not necessarily lead to premature dropout or poorer engagement [54]. Notwithstanding these positive treatment prognoses, TC providers should try to monitor psychological distress throughout the entire treatment process since this may still present a barrier to change. Eventually, early identification of psychological problems may help clients to overcome obstacles more quickly, which in turn may speed up the recovery process.

### Determinants of psychological development and change

With respect to the second DCI dimension, Personal Development and Change, the results indicated fixed variables as significant predictors. The strongest predictor was judicial referral. It was shown that clients who were referred to treatment by the criminal justice system – as a condition of probation or parole – reported higher Personal Development and Change scores than clients who entered treatment voluntarily or who entered following advice from family, friends or professionals. Although a judicial referral can be viewed as an external motivation to enter treatment, it does not explain the higher Personal Development and Change scores. In a sense, our results contradict earlier research findings, particularly those studies that found poorer levels of internal motivation and engagement in criminal justice clients than in non-criminal justice clients [55,56]. What might be the cause for higher Personal Development and Change scores in our study is the threat and fear for imprisonment combined with the chance to change their life courses. For instance, in a qualitative study by Colman, De Wree & De Ruyver [57] on the application of alternative sanctions for drug offenders, participants reported a positive reassessment of life and improved personal insight due to a judicial intervention. In particular, the combination of substance abuse treatment and a judicial referral was highly appreciated by most of the interviewees [57]. Although the findings in our study could be viewed as an indication that judicial referral to TC treatment does not necessarily mean poorer treatment prognoses, socially desirable answers might also have caused higher Development and Change scores.

Previous findings have considerable implications for TC treatment in general. Clients’ suitability for treatment and their ability to connect with others in the program are necessary requirements for TC treatment to be successful. Also, meta-cognitive abilities such as insight and self-reflection are considered prerequisites for lasting recovery. Although the TC environment is specifically developed to stimulate incremental multidimensional learning, the effects only start to occur after the third month of treatment [58]. Building on the empirical evidence that most clients leave treatment within these first three months [7,9], it seems important that the recovery process is stimulated earlier in the TC treatment process. Therefore, it seems essential to establish a profile of clients’ suitability, psychological well-being and personality features already at intake and to keep monitoring further developments, even beyond treatment completion. Also, the implementation of cognitive learning strategies at the beginning of treatment could help improve clients’ (meta-)cognitive functions and eventually speed up the recovery process.

### Limitations of the study

The present study has some limitations. First, all data was self-reported and as such were subject to the limitations of self-report data in general. Second, our study was a cross-sectional study and thus only provided a snapshot of the association between client characteristics and the treatment process. To better distinguish whether motivation or treatment distress precede or follow changes in the treatment process, a longitudinal study is needed. Third, the sample size was relatively small which may have increased the risk for type II errors. It should be noted that due to limited variation in some of the independent variables (i.e. race, gender, marital status, prior drug treatment, EuropASI composite scores), the effects may not have been detected at a level that reached statistical significance. However, when comparing the subject’s background characteristics with the characteristics of another study [59], we found them to be very similar. This indicates that the results may be generalized, at least for Flemish TC residents. We would like to emphasize that we have dealt with personality traits (Cluster A, B ad C) as conditions that are stable over time. We are aware of recent empirical studies that have indicated that the stability of the disorder constructs is considerably lower than implied by the DSM-IV. We know that normal and pathological personality traits may change across the lifespan, but given the use of the ADP-IV as a measure of DSM-IV personality disorders, we considered personality traits as fixed variables in this study. Finally, it should be noted that while evidence exists for the eight DCI subscales [28,60], we only performed regression analyses on the two summary dimensions which appeared to have a higher degree of internal consistency.

## Conclusions

The present study is the first to explore the relationship between both fixed and dynamic client factors and the TC treatment process, using a multidimensional, TC-specific instrument. We found that during the first month of treatment, dynamic client variables more strongly affected clients’ perceptions of the TC environment than fixed client variables. Clients’ views on Personal Development and Change was solely related to fixed client characteristics. The results suggest the need for a greater attention to clients’ psychological well-being, the presence of personality disorders (especially ‘Cluster B’) and intergenerational drug/alcohol use in order to enhance clients’ early engagement in the TC environment. Special attention should be devoted to treatment suitability as part of the motivational assessment as this seems to be a very strong predictor of how clients react to the initiation stage of TC treatment. Given that the overall ratings of clients on Personal Development and Change are low, the authors suggest the introduction of (meta-)cognitive training strategies in the pre-program stage and/or the induction stage of TC programs. These strategies could help improve clients’ meta-cognitive skills needed to achieve insight and self-reflection and perhaps speed up the process of recovery.

## Competing interests

The authors declare that they do not have any competing interests.

## Authors’ contributions

IG conceived this study and was responsible for the research design, the data collection and statistical analysis and the manufacture of the manuscript. WV helped with the statistical analysis and with the draft of the manuscript. SV assisted with the manufacture of the manuscript and EB participated in the design and helped with the draft of the manuscript. All authors read and approved the final manuscript.
